# Trusting relationships between patients with non-curative cancer and healthcare professionals create ethical obstacles for informed consent in clinical trials: a grounded theory study

**DOI:** 10.1186/s12904-023-01204-6

**Published:** 2023-07-01

**Authors:** Mary Murphy, Eilís McCaughan, Gareth Thompson, Matthew A Carson, Jeffrey R Hanna, Monica Donovan, Richard H Wilson, Donna Fitzsimons

**Affiliations:** 1 Resuscitation Services, Elliott Dynes Building Royal Victoria Hospital, Belfast Health and Social Care Trust, Belfast, UK; 2 School of Nursing and Midwifery Institute of Nursing and Health Research, Ulster University, Coleraine, UK; 3 School of Nursing and Midwifery Medical Biology Centre, Queen’s University Belfast, Belfast, UK; 4 Institute of Cancer Sciences, University of Glasgow, Glasgow, UK

**Keywords:** Clinical trial participation, Patients with non-curative cancer, Healthcare professionals, Decision-making, Consent process, Qualitative study, Grounded theory, Interviews

## Abstract

**Background:**

Clinical trial participation for patients with non-curative cancer is unlikely to present personal clinical benefit, which raises the bar for informed consent. Previous work demonstrates that decisions by patients in this setting are made within a ‘trusting relationship’ with healthcare professionals. The current study aimed to further illuminate the nuances of this relationship from both the patients’ and healthcare professionals’ perspectives.

**Methods:**

Face-to-face interviews using a grounded theory approach were conducted at a regional Cancer Centre in the United Kingdom. Interviews were performed with 34 participants (patients with non-curative cancer, number (*n*) = 16; healthcare professionals involved in the consent process, *n* = 18). Data analysis was performed after each interview using open, selective, and theoretical coding.

**Results:**

The ‘Trusting relationship’ with healthcare professionals underpinned patient motivation to participate, with many patients ‘feeling lucky’ and articulating an unrealistic hope that a clinical trial could provide a cure. Patients adopted the attitude of ‘What the doctor thinks is best’ and placed significant trust in healthcare professionals, focusing on mainly positive aspects of the information provided. Healthcare professionals recognised that trial information was not received neutrally by patients, with some expressing concerns that patients would consent to ‘please’ them. This raises the question: Within the trusting relationship between patients and healthcare professionals, ‘Is it possible to provide balanced information?’. The theoretical model identified in this study is central to understanding how the trusting professional-patient relationship influences the decision-making process.

**Conclusion:**

The significant trust placed on healthcare professionals by patients presented an obstacle to delivering balanced trial information, with patients sometimes participating to please the ‘experts’. In this high-stakes scenario, it may be pertinent to consider strategies, such as separation of the clinician-researcher roles and enabling patients to articulate their care priorities and preferences within the informed consent process. Further research is needed to expand on these ethical conundrums and ensure patient choice and autonomy in trial participation are prioritised, particularly when the patient’s life is limited.

## Background

The advancement of cancer treatment is contingent on research that investigates novel drugs and treatment methods, with the primary phase of this process constituting a clinical trial [1]. Clinical research studies are governed by stringent scientific procedures and regulations to maximise patient protection and enhance the rigour of findings, with one of these principles being informed consent, which allows potential participants to make a voluntary, autonomous decision regarding trial enrolment, through the provision of comprehensible information surrounding the potential benefits and risks of involvement [2]. Additionally, patients should be informed of alternative opportunities to study participation, for instance, supportive care within a palliative setting [3]. However, the decision-making process for participation in an oncology clinical trial inevitably occurs when patients are vulnerably positioned, due to the life-threatening circumstances of a non-curative cancer diagnosis, raising concerns about the validity of informed consent provided [4].

Research regarding clinical trial decision-making and informed consent has been predominantly performed in curative settings, with a paucity of studies focusing on the palliative context in patients with non-curative cancer nearing the end of life [1]. In this patient population, early phase trials are unlikely to present clinical benefit [5]. Thus, the decision to participate represents an ethically challenging and clinically complex situation [4]. Research in this area asserts that a desire for curative treatment constitutes the principal reason for patients with non-curative cancer participating in clinical trials [6], with many patients being willing to try anything to improve prognosis [7]. These findings are supported by a grounded theory study recently published by our group [8], which identified ‘Nothing to lose’ as the core category that underpinned the decision by patients with non-curative cancer to participate in clinical trials. Moreover, this participation was regarded by patients as the ‘only hope in the room’, with the decision executed within a ‘trusting relationship’ with healthcare professionals. Ultimately, these findings suggest that patients with non-curative cancer frequently hold unrealistic hopes for personal benefit from clinical trials, which highlights a requirement for research to develop a more robust and context appropriate consent process.

Decisional aids have been developed to support patients with cancer in the consent process for clinical trial participation, such as: websites [9], booklets [10], and videos [11]. However, a recent systematic review reported limited evidence for the effectiveness of these decisional support resources, with insufficient research on interventions that account for the patients’ relationship with clinical staff [12]. A better understanding of the impact of this relationship on clinical trial participation would inform intervention development for supporting patients during the decision-making and informed consent process, thereby maximising patient protection and enhancing quality of care. Given the relevance of our recent qualitative findings [8], this paper seeks to further explore the ‘trusting relationship’ between patients with non-curative cancer and clinical staff, to better understand how the nuances of this relationship may impact the decision-making and informed consent process from both perspectives.

## Methods

### Study aim

To develop a greater understanding of how the healthcare professional-patient relationship impacts decision-making and informed consent for clinical trial participation in patients with non-curative cancer, from the perspectives of the patients and healthcare professionals involved in the consent process and recruitment procedures.

### Study design

The study was performed at a regional Cancer Centre (Northern Ireland Cancer Centre, Belfast Health and Social Care Trust) in the United Kingdom (UK), which provides standard cancer treatment to patients from across Northern Ireland. Face-to-face interviews were conducted with participants. The overarching methodology was grounded theory, which is a suitable approach for generating theoretical explanations to elucidate understudied areas related to personal experience, behaviour, and concerns [13].

### Participants

Sixteen patients with non-curative cancer and eighteen healthcare professionals involved with the consent process and recruitment procedures were recruited from the collaborating regional Cancer Centre in the UK. Eligibility criteria for participants are presented in Table 1.

**Table 1:** Eligibility criteria

Patients	
Inclusion Criteria	Exclusion Criteria
Previous invitation to participate in a clinical trial.	< 18 years of age.
Non-curative solid tumours or non-curative haematological malignancy.	A poor understanding of spoken English.
> 12 weeks and < 5 years projected survival.	
**Healthcare Professionals**	
**Inclusion Criterion**	
Identified by the patient as contributing to their consent related decision-making or trial recruitment, including both research nurses and doctors.	

ey: Oval = category; Speech bubble = participant quote; Orange = category 1 (core category); Yellow = category 2; Green = category 3; Grey = category 4; Blue = category 5; P = patient; HcP = healthcare professional.

### Recruitment

The recruitment strategy involved a convenience sampling approach for patients, whereby oncologists identified eligible patients and outlined the study to them. If a patient expressed interest, the oncologist provided him / her with the participant information sheets and requested verbal agreement for the patient to be contacted by a researcher (MM) for questions to be answered and an interview arranged. Theoretical sampling was utilised for healthcare professionals following the analysis of patient data as patients frequently reported oncologists and nurses as being influential in their decision-making process for clinical trial participation, which enabled elucidation of emergent core categories. Healthcare professionals were invited to participate via an email from the researcher (MM), which contained an overview of the study and participant information sheets. Written informed consent was obtained from all participants prior to interviews by the researcher (MM).

### Interviews

Interviews were conducted by MM, a female healthcare professional and researcher with training in interviewing skills and grounded theory, who had no prior relationship with patient participants and a minimal relationship with healthcare professionals (awareness of each other’s professional capacity). Consistent with the emergent nature of grounded theory, a formal interview guide was not used [13]. Rather, interviews were initiated with a general request, such as ‘Tell me about when you were first diagnosed with cancer?’. Follow-up questions were conceived during each interview and were determined by and relevant to the participants’ responses (i.e., ‘How did you feel when you received your diagnosis/prognosis?’, ‘When was a clinical trial first mentioned?’, and ‘Why did you make your decision about participation?’). Patient interviews were conducted at their preferred location, which included the patient’s own home (*n* = 13) or a private room at a hospital (*n* = 3). Healthcare professional interviews were facilitated in a quiet room on hospital premises (*n* = 18). All interviews lasted between 40 and 80 min (average: 60 min).

### Data analysis

Interviews were audio-recorded and transcribed verbatim. Data analysis was performed iteratively, which aligns with the constant comparative method [14]. As per grounded theory techniques [15], the analysis comprised open coding, selective coding, and theoretical coding. Initial transcripts were coded by MM, DF, and EMcC before identified codes were compared. As the analysis progressed, the identified codes were reviewed and refined by the research team through critical dialogue until consensus agreement. Coding was completed by hand as the use of NVivo made the researchers feel ‘distanced’ from the data. Pseudonyms replace any names in participant quotes to uphold anonymity.

## Results

A total of 34 participants were involved (patients, *n* = 16; healthcare professionals, *n* = 18). The patient sample were predominantly male (*n* = 11), had an average age of 57.6 years, exhibited a range of cancer types (i.e., breast, oesophageal, pancreatic, or prostate), and data subsequently obtained from medical records confirmed that patients were interviewed shortly before death (range: 7–64 weeks). Three patients declined clinical trial participation, with thirteen patients primarily participating in a Phase III Clinical Trial. Regarding the healthcare professional sample, they were predominantly female (*n* = 10) and comprised Oncologists (*n* = 10) and Clinical Research Nurses (*n* = 8). Participant characteristics have been comprehensively reported elsewhere [8].

### Category 1 (core category) – trusting relationship

**Fig. 1:**
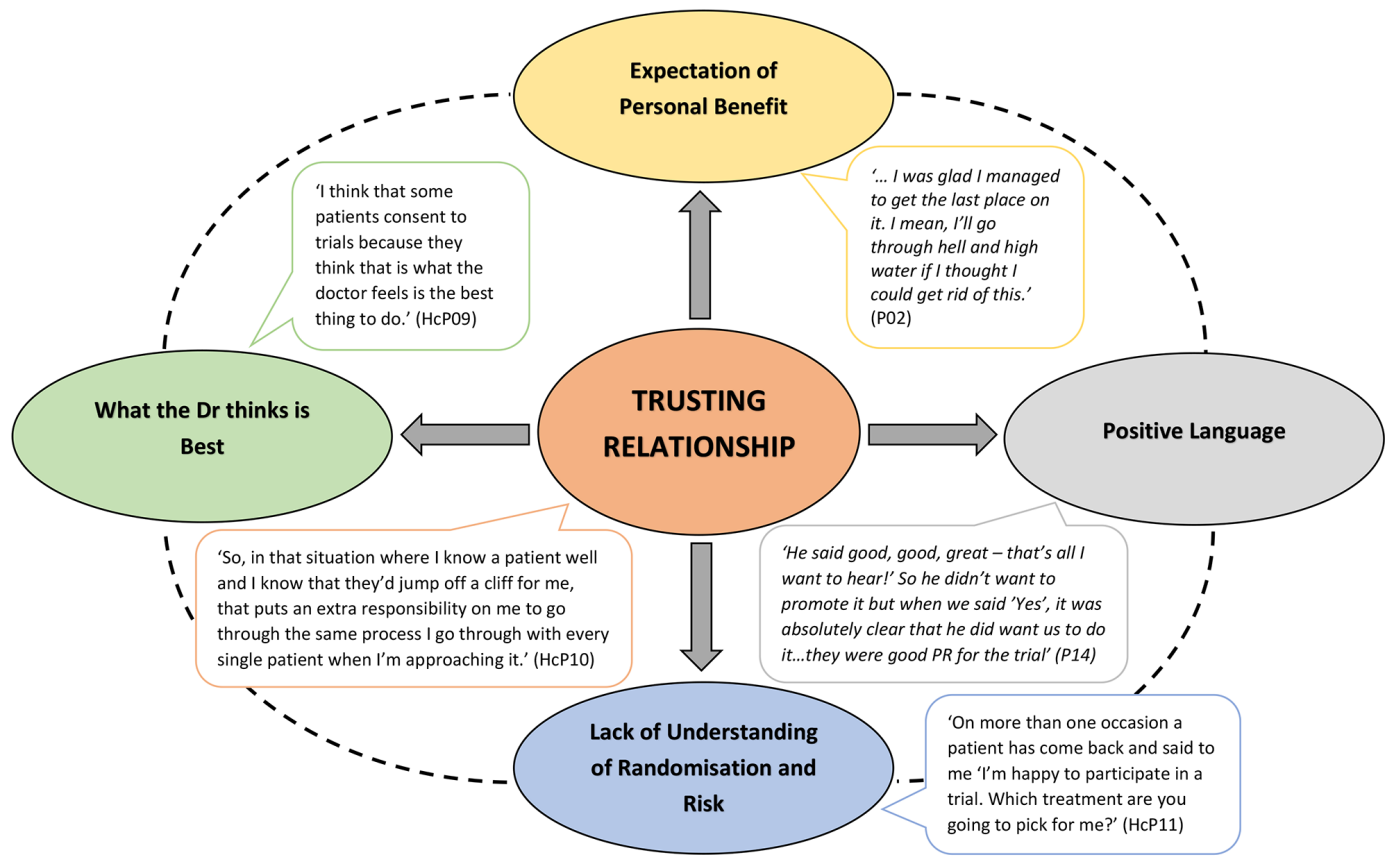
Visual representation of identified categories

>The ‘Trusting relationship’ was identified as a core category for both patients with non-curative cancer and healthcare professionals involved with the consent process and recruitment procedures (see Fig. 1). Many healthcare professionals reflected on their experience and mentioned the challenges of maintaining a neutral stance when discussing clinical trial participation due to this trusting relationship with patients:

*‘So, in that situation where I know a patient well and I know that they’d jump off a cliff for me, that puts an extra responsibility on me to go through the same process I go through with every single patient when I’m approaching it.’* (HcP10).

Most healthcare professionals believed they presented information in a balanced way to patients about inclusion in a clinical trial, with the aim of facilitating an informed decision regarding clinical trial participation:

*‘If they ask me an honest question about what I think their personal chance of gain from this is – I’ll answer that honestly - if you are entering a Phase I clinical trial, you have a very small chance of clinical benefit and if I was in that situation and the patient asked, “What would you do doctor?” I would not do it, because I see a lot of down sides in it for them. This is somebody who is dying, who has got a limited life expectancy and who we are asking to spend a lot of their time, a significant amount of their limited time, having pharmacokinetic studies carried out on them.’* (HcP15).

However, the trial information was not always perceived in the same balanced way by patients:

*‘But he did say great things on that day. He said that XX was a drug that cost £30k or £40k a course. It works well, but this other one, the trial drug is even better than XX he thinks, or they think it’s even better.’* (P14).

Within the trusting relationship, patients respected and valued the expertise of healthcare professionals. As a result, healthcare professionals believed that patients were determined to ‘please them’ by participating in a clinical trial:

*‘I think that there is a risk that patients might feel, “Well I want to keep in with Dr Smith, he’s my oncologist and it’s very important for me. And how do I keep Dr Smith sweet? Well then, I keep Dr Smith sweet by doing trials.”’* (HcP06).

It is evident that there are many challenges for healthcare professionals when providing patients with balanced trial information because of their emotionally charged relationship. While healthcare professionals believed that they did their best to present impartial information, patients perceived that the doctor wanted them to join the clinical trial, which may have resulted in decisions to participate to please the healthcare professionals. The core category of ‘Trusting relationship’ overarched the data and was inextricably linked to the other categories, which are discussed below.

### Category 2 - expectation of personal benefit

Most patients exhibited an ‘Expectation of personal benefit’ (i.e., extended life) from trial participation:

*‘I felt pleased. It’s very hard to define. I did feel pleased. I felt very positive about it. I was glad I managed to get the last place on it. I mean, I’ll go through hell and high water if I thought I could get rid of this. I’d like a few more years.’* (P02).

Patients were sometimes aware of the expensive cost of trial drugs and thought this represented superior treatment in comparison to standard care:

*‘It’s not available on the NHS [National Health Service], you pay for it yourself and I think it’s £3,000 per treatment, so it works out at £30,000 a year if you wanted to pay for the treatment. So, I felt I was lucky enough getting this treatment without having to pay for it cos I couldn’t have afforded it naturally you know and so hopefully I will get a bit of benefit from it.’* (P09).

The offer of a clinical trial was clearly perceived as something beneficial by patients, despite the non-curative intent. This demonstrates evidence of a strong, almost desperate desire to extend life, with some patients disclosing a hope that clinical trial participation would be a “magic bullet”, and against all odds, they would be cured.

### Category 3- what the doctor thinks is best

Secondary to the ‘Trusting relationship’ demonstrated in category 1, patients displayed an attitude of ‘What the doctor thinks is best’. It was evident from many patient accounts that they were keen to take the advice of their doctor in making the decision about trial participation:

*‘I didn’t think to bother to question it because I just took the view that they’re not going to give me something that’s absolutely stupid. I trust them and I’m not going to question this.’* (P02).

Also, ‘Doing what the doctor thinks is best’ was thought by the healthcare professionals to have an influence on a patient’s decision-making:

*‘I think that some patients consent to trials because they think that is what the doctor feels is the best thing to do.’* (HcP09).

This degree of trust placed the healthcare professionals in a difficult position when discussing clinical trial participation with patients, with many patients expecting healthcare professionals to make the decision on their behalf by virtue of their ‘expert status’, which may have compromised the impartiality of information provided:

*“Any attempt to enter into a more equal conversation unsettles them and is unhelpful to the patient. Frequently you are asked “what would you do if it was you, Doctor?” or “Tell me what’s right, you are the expert.”* (HcP10).

This analysis demonstrates that given the life-or-death situation, patients placed a lot of trust in their doctor and his / her expertise and judgement, and as a result, many patients were keen to take up the trial offer.

### Category 4 - positive language

During interactions between patients and healthcare professionals, there was evidence that the language and demeanour used to describe the trial had a powerful impact. This patient is recalling the conversation with his Oncologist, when he agreed to be a trial participant:

*‘He [oncologist] said, “Good, good, good, great, that’s all I want to hear.” So, you know, he didn’t want to promote it, but when we said, “Yes,” it was absolutely clear, he did want us to do it. And Dr Black was very positive about it. I didn’t feel pressure… No pressure, but em, they were good PR for the trial. Just very positive about the trial.’* (P14).

The healthcare professionals shared this perception and believed the environment had a positive impact on patients’ decision-making:

*‘And attending a clinic where research is going on, a research active clinic, I would think patients get confident like, “Well these boys know what they are doing. If these doctors and nurses are promoting this type of research, they probably know what they are doing.” And they’re right.’* (HcP10).

Ultimately, patients were keen to enroll if healthcare professionals exhibited positive views on clinical trial participation, which reflects the degree of influence conferred by their trusting relationship with patients.

### Category 5 - lack of understanding of randomisation and risk

Data from both patients and healthcare professionals strongly suggested that patients found it difficult to understand some important elements of the consent process (i.e., randomisation) and believed the healthcare professionals would choose the drug regime to which they would be allocated. This misunderstanding undermined informed consent, with many patients being ‘naïve’ to the limitations of allocation to a control group (i.e., loosing time with loved ones):

*‘I was also told that the selection for whichever arm is being done by computer random selection. But having said that, the selection process is a matter of putting information into a computer of a patient’s condition and then the computer will say this will be more appropriate. At the end of the day, and taking into account the high symptom score that I got, would tend to influence a decision for an arm of the research that would be at the more severe end of the scale.’* (P04).

Healthcare professionals understood this difficulty:

*‘On more than one occasion a patient has come back and said, “I am happy to participate in a trial. Which treatment are you going to pick for me?”.’* (HcP11).

Despite having been given information that clearly presented the possibility of potentially fatal consequences, the view of most patients was:

*‘What’s an adverse reaction to getting rid of this?’* (P02).

In all accounts, the high-risk environment of an incurable disease exerted a powerful influence on decision-making, with the enormity of the diagnosis eliciting a naïve belief for patients that their relationship with healthcare professionals would favourably influence study treatment (i.e., ensuring allocation to the treatment group). Ultimately, this resulted in under appreciation of the risks involved with clinical trial participation, which compromised the integrity of informed consent.

## Discussion

This study is one of the first to explore how the trusting healthcare professional-patient relationship impacts decision-making and informed consent for clinical trial participation in patients with non-curative cancer. The theoretical model generated by the grounded theory analysis is presented in Fig. 1. The core of this model is ‘Trusting relationship’, which encapsulates and influences the other identified categories. This model builds on previous data [8] by elucidating the impact of the trusting healthcare professional-patient relationship on the decision-making process for clinical trial participation. From the data, it is apparent that the ‘Trusting relationship’ between patients and healthcare professionals resulted in patients doing ‘What the Doctor thinks is best’ and placing a huge amount of trust in the healthcare team by virtue of their expertise. In turn, this made it difficult for healthcare professionals to deliver balanced information, with patients deciding to participate to please the ‘expert’ healthcare professionals, along with naïvely believing the healthcare professionals would favourably influence their clinical trial treatment. Ultimately, the emotive healthcare professional-patient relationship resulted in patients under appreciating the risks involved with clinical trial participation, which may compromise the integrity of informed consent.

To uphold autonomy, patients with non-curative cancer should not be excluded from clinical trials based on vulnerability [16]. However, the categories identified in this study outline ethical complications in the decision-making and informed consent processes for clinical trial participation in this patient population. Firstly, patients with non-curative cancer exhibited unrealistic expectations of personal benefit, which raises concerns about the validity of informed consent provided. Consistent with the principles of ‘therapeutic misconception’ [17], a potential explanation for this patient behaviour is an inability to differentiate between research follow-up and standard care, with a misunderstanding that the primary aim of research is to care for them [18].

Given the high stakes involved and the role overlap between clinician and researcher, the question presented by the findings of this study is: ‘Is consent truly informed in this situation?’. To avoid this ‘ethical fading’, a psychological process involving ethical aspects of a decision disappearing from view when focusing on other aspects (i.e., perceived benefits) [19], healthcare professionals and researchers should consciously acknowledge the vulnerability and desperation for ‘a cure’ of patients with non-curative cancer when discussing clinical trial participation [8]. To maximise patient autonomy and the integrity of informed consent, healthcare professionals must employ an effort to ensure patients are impartially informed, both in writing and verbally, of the scope and aims of clinical trials prior to invitation, whilst avoiding any preformed decisions that exclude patient involvement [20]. Future research should explore these issues from the perspectives of healthcare professionals, for instance, opinions on fulling a ‘supportive’ rather than ‘curative’ role [21].

Shared decision-making is a core value of cancer care [22], yet the findings of this study question the degree to which patients are serving as active partners in the decision-making process. Informed consent discussions for participation in Phase-I clinical trials mostly occur in hospital-based clinics involving members of the clinical research team. As demonstrated by the results of this study, the emotive relationship between patients and the clinical research team resulted in patients deciding to participate to please the ‘experts’. To maximise patient autonomy and integrity of informed consent, a multidisciplinary approach with distributed deliberation is required, whereby patients discuss options with those independent of the clinical research team at different times and places [23]. Importantly, the recognition of this patient requirement and allowing time for it, are essential for effective shared decision-making [24]. Greater collaboration with and inclusion of palliative care clinicians would optimise the level of information provided to patients in this setting, such as: expert discussion of prognosis and options for supportive care [25]. To facilitate this, effective communication between the oncologists and palliative care specialists is required, with the wishes of patients and families systematically assessed and integrated into the decision-making processes [26].

In addition to palliative care clinicians, there is a need for greater involvement of primary care providers in the decision-making process for clinical trial participation, with General Practitioners (GPs) being well placed to support patients during their cancer care pathway by virtue of established therapeutic relationships [27]. Of all caregivers involved, GPs are best positioned to balance treatment options according to a patient’s medical history and personal preferences [28]. Indeed, recent studies have shown that patients are motivated to consult their GP in preparation for final treatment decisions with their oncologist [29, 30]. Whilst there are logistical challenges to this, adequate planning is required to include such consultations in the decision-making process. Ultimately, there is a need to rethink the cancer pathway for patients with non-curative disease to promote better collaboration with other healthcare professionals within and across all levels of care, which in the context of clinical trial participation, may improve the integrity of informed consent and alleviate the difficulty of delivering ‘balanced information’ perceived by the clinical research team.

In terms of strengths and limitations, the current study adds depth to previous work by illuminating how the healthcare professional-patient relationship impacts decision-making and informed consent, with the holistic inclusion of patients and healthcare professionals involved in these processes. By implementing a grounded theory approach, the study provides an important contribution to the literature by appropriately elucidating an understudied area. However, despite these strengths, the research was conducted in a small sample who were predominantly male (*n* = 11) from one cancer centre in the UK, therefore, the transferability of the findings to other settings is not assured. There is a need for wider exploration of these issues in a larger multi-centre study with a more diverse gender population, which would inform applicability of the theoretical model to cancer patients and relevance to other diseases (i.e., end-stage renal disease and heart failure). Moreover, the literature would benefit from a better understanding of the perspectives of trial decliners and caregivers, which would provide alternative perspectives and insights into involvement in the decision-making process.

## Conclusion

This study has expanded understanding of how the trusting healthcare professional-patient relationship can influence decision-making and informed consent in patients with non-curative cancer. The categories identified in this study convey serious ethical complications, with patients placing a degree of trust on healthcare professionals that rendered the delivery of balanced trial information difficult. As a result, patients often believed that deciding to participate would please the ‘experts’. Healthcare professionals must acknowledge the vulnerability of patients in this setting, whilst avoiding preformed decisions and delivering unbiased study information. To assist with this, a multidisciplinary approach with distributed deliberation would enable other professionals, without direct care responsibility, to lead informed consent discussions, which may facilitate effective shared decision-making with patients. Given that standard care and research are becoming increasingly interwoven, future research should further explore the ethical conundrums identified by this study. The imperative is to promote a clinical environment that upholds ethical research, thereby maximising patient autonomy and enhancing quality of care.

## Data Availability

The dataset analysed during the current study is not publicly available given that data was collected from one clinical setting, meaning there is potential for professionals to be identified based on the nature of their comments. As such, we do not have ethical approval to upload full transcripts. However, given the extensive excerpts used we feel there is appropriate information on this data in the public domain. Further information is available from the corresponding author on request.

## List of abbreviations

UK: United Kingdom
P: Patient
HcP: Healthcare professional
GP: General Practitioner
